# Predicting Microbial Protein Synthesis in Cattle: Evaluation of Extant Equations and Steps Needed to Improve Accuracy and Precision of Future Equations

**DOI:** 10.3390/ani14192903

**Published:** 2024-10-09

**Authors:** Michael L. Galyean, Luis O. Tedeschi

**Affiliations:** 1 Department of Veterinary Sciences, Texas Tech University, Lubbock, TX 79409-2123, USA; 2 Department of Animal Science, Texas A&M University, College Station, TX 77843-2471, USA

**Keywords:** cattle, energy intake, microbial crude protein synthesis, prediction equations, resampling

## Abstract

**Simple Summary:**

Accurate and precise predictions of microbial crude protein (MCP) synthesis are crucial to predicting the supply of metabolizable protein in cattle. Inaccurate estimates of MCP can lead to over- or underfeeding of protein in beef cattle diets, which can have important animal performance and economic consequences. Extant MCP prediction equations are generally based on the intake of energy (e.g., total digestible nutrients (TDN) or digestible energy) and protein. We developed new equations based on the intake of organic matter, which yielded similar fit statistics to the extant equations for observed vs. predicted values, but the precision of all MCP predictions was less than desired, with errors averaging more than 28% of the observed mean. A simple approach of calculating MCP as 10% of the TDN intake was as effective as more complex equations. To move forward and improve the accuracy and precision of MCP prediction equations, research is needed to develop consistent and more precise techniques to measure MCP synthesis in cattle that will yield reliable estimates across a wide range of diets and production situations. Meta-omics tools might be a useful component of these new methods, but additional research is needed.

**Abstract:**

Predictions of microbial crude protein (MCP) synthesis for beef cattle generally rely on empirical regression equations, with intakes of energy and protein as key variables. Using a database from published literature, we developed new equations based on the intake of organic matter (OM) and intakes or concentrations of crude protein (CP) and neutral detergent fiber (NDF). We compared these new equations to several extant equations based on intakes of total digestible nutrients (TDN) and CP. Regression fit statistics were evaluated using both resampling and sampling from a simulated multivariate normal population. Newly developed equations yielded similar fit statistics to extant equations, but the root mean square error of prediction averaged 155 g (28.7% of the mean MCP of 540.7 g/d) across all equations, indicating considerable variation in predictions. A simple approach of calculating MCP as 10% of the TDN intake yielded MCP estimates and fit statistics that were similar to more complicated equations. Adding a classification code to account for unique dietary characteristics did not have significant effects. Because MCP synthesis is measured indirectly, most often using surgically altered animals, literature estimates are relatively few and highly variable. A random sample of individual studies from our literature database indicated a standard deviation for MCP synthesis that averaged 19.1% of the observed mean, likely contributing to imprecision in the MCP predictions. Research to develop additional MCP estimates across various diets and production situations is needed, with a focus on developing consistent and reliable methodologies for MCP measurements. The use of new meta-omics tools might improve the accuracy and precision of MCP predictions, but further research will be needed to assess the utility of such tools.

## 1. Introduction

Predictions of microbial crude protein (MCP) synthesized in the rumen are critical to the application of current protein requirement systems for beef cattle [1]. Because protein is often an expensive supplemental nutrient, inaccurate estimates of MCP synthesis have important practical consequences in ruminant feeding. Nonetheless, extant MCP prediction equations are characterized by relatively large prediction errors (20 to 30% of the observed mean [2,3]).

The prediction of MCP synthesis is complicated by the limited data available for the development and evaluation of equations. Because MCP cannot be measured directly in the digesta of cattle consuming typical feedlot and grazing diets, estimates of MCP synthesis in cattle have traditionally relied on invasive surgical procedures to install intestinal cannulas and markers to estimate both digesta flow and MCP concentration in the digesta flow. An alternative approach has been the use of purine derivatives in the urine, particularly allantoin and uric acid when the method is applied to cattle [4]. Total urine collection is optimal, but many studies in the literature have used spot urine samples, with total urine flow calculated assuming a constant excretion of creatinine in the urine relative to body weight. Although general agreement with estimates derived from intestinal sampling has been demonstrated, MCP estimates from urinary purine derivatives are generally considered relative rather than absolute estimates [4,5].

In this synthesis and review paper, we develop new MCP prediction equations and evaluate these equations along with extant prediction equations using resampling and a simulated multivariate normal (MVN) population derived from a literature database of 335 observations reported by [2]. We then consider options for improving current empirical prediction equations and the development of new approaches to predict MCP synthesis in cattle.

## 2. Equation Development and Evaluation of Extant Equations

### 2.1. Database and Development of New Equations

The database used by [2] to develop MCP prediction equations based on the intakes of dietary energy and crude protein (CP) was used to examine the possibility of developing additional equations. Details regarding the selection of studies for the database are provided in previously published reports [2,6]. Because the intake of dry matter (DM) and organic matter (OM) are highly related to MCP synthesis [6], we were interested in determining the value of OM intake combined with the intakes or concentrations of various dietary components. Initially, the PROC REG of SAS (SAS Inst. Inc., Cary, NC, USA; version 9.3) was used to screen relationships between MCP and the intake of OM and intakes or dietary percentages of CP, starch, ether extract, and neutral detergent fiber (NDF). A stepwise, forward selection process was specified, with an entry-level *p*-value of 0.10, to determine which variables were most highly predictive of MCP synthesis. The organic matter intake was the first variable selected in both models, but when intakes of CP, starch, ether extract, and NDF were included in the model, only the intakes of CP, NDF, and starch met the model entry requirement (i.e., *p* < 0.10). Nonetheless, because the intake of starch increased the model R^2^ by less than 1%, only the intakes of CP and NDF were used in the subsequent analyses. In the model using dietary concentrations as potential variables for selection, only CP and NDF met the model entry criterion of *p* < 0.10.

Subsequently, PROC MIXED of SAS was used to develop MCP synthesis prediction equations based on the OM intake and the intakes or dietary percentages of CP and NDF. The models included random intercept effects with the “study” factor as the subject and an assumed unstructured covariance structure.

As a simple method of predicting MCP synthesis, we also considered an approach akin to the NRC [7] prediction equation, whereby the MCP is calculated as the intake of TDN multiplied by 0.13, which was based on the work of [8], with an additional adjustment for diets with dietary physically effective NDF (peNDF) values less than 20% that was based on the work of [9]. Instead, we chose a value of 0.10 multiplied by the TDN intake, which is the average for grams of MCP per gram of the TDN intake in the Galyean and Tedeschi [2] database, and we did not include an adjustment for peNDF.

### 2.2. Evaluation of Newly Developed and Extant Equations

The predicted and observed values for the two newly developed equations based on the intake of OM, the intakes or percentages of CP and NDF, and the simple equation using 10% of the TDN intake to predict MCP, were evaluated along with several extant equations. The extant equations included the NASEM [1] equation based on the TDN intake, the TDN intake and the TDN intake + CP intake equations of [2], and the BR-CORTE equation [10] based on the TDN and CP intakes. These equations are shown in Table 1.

#### 2.2.1. Resampling Analyses for Equation Evaluation

The observed and predicted values from the Galyean and Tedeschi [2] dataset for each equation shown in Table 1 were sampled with a replacement using PROC SURVEYSELECT of SAS to yield 500 resamples of the original dataset to evaluate the goodness-of-fit statistics of prediction equations. For each resampling, PROC IML of SAS was used to determine the coefficient of determination and root mean square error of prediction (RMSEP), with the concordance correlation coefficient (CCC) computed as described by [11]. In addition, the mean squared error of prediction (MSEP) was decomposed by determining the relative contributions of mean bias, slope bias, and random error and expressing them as a percentage of the MSEP [12]. The PROC MEANS and PROC STDIZE of SAS were then used to compute the average and the 5% and 95% quantiles for the resampled fit statistics data.

#### 2.2.2. Simulating a MVN Population for Equation Evaluation

As an alternative approach to resampling, we simulated an MVN population from the original Galyean and Tedeschi [2] database. The variables included in the simulated population were average animal body weight (kg), yield of MCP (g/d), intakes (g/d) of DM, OM, ruminally degraded protein, TDN, CP, ether extract, NDF, and acid detergent fiber, as well as the intake of digestible energy (Mcal/d). A correlation matrix for these variables was created using PROC CORR of SAS, which was then used in PROC SIMNORMAL of SAS to create 500 samples of 335 observations each, yielding the same number of total observations (167,500) as the resampling analysis. For each simulated sample, the RMSEP, CCC, decomposition of the MSEP, and 5% and 95% quantiles were calculated using PROC IML of SAS as described for the resampling analyses.

## 3. Equation Evaluation Results

### 3.1. Newly Developed Equations

Of the seven equations evaluated, three are new equations (Equations (1) through (3) in Table 1) derived from the literature database of [2]. The details of fit statistics for Equations (1) and (2) are provided in the footnotes of Table 1. Because Equation (3) was not derived from regression but merely reflected the average ratio of MCP synthesis to the TDN intake in the literature database, no regression fit statistics were available.

Equations (1) and (2), which are based on the OM intake and either intakes (Equation (1)) or percentages (Equation (2)) of CP and NDF, yielded overall fit statistics (e.g., root mean square error and R^2^) similar to those reported by [2] for the equations based on intakes of TDN or the TDN and CP intake shown in Table 1. This finding was expected, as [6] reported a strong relationship between MCP synthesis and the DM intake, presumably reflecting the fact that intakes of DM or OM are reasonably strong proxies for fermentable OM, at least in the Galyean and Tedeschi [2] database. Using a different database, Hanigan et al. [3] also reported a strong relationship between the DM intake and the MCP outflow from the rumen and noted the collinearity between the DM intake and intakes of various nutrients. Thus, although regression fit statistics were similar for the two equations, the use of Equation (2), which used percentages of CP and NDF vs. intakes of these nutrients in Equation (1), might decrease concerns about multicollinearity.

### 3.2. Evaluation of New and Extant Equations

All seven equations in Table 1 were evaluated by resampling or by application to simulated MVN populations, with results shown in Table 2.

Results for the resampling and simulated MVN population samples were not appreciably different, so a general discussion of equation fit statistics will apply to both evaluation approaches. Perhaps not surprisingly, the equations that were developed from the Galyean and Tedeschi [2] database (the TDN and TDN + CP intakes and Equations (1) and (2) in Table 1) yielded similar fit statistics. Likewise, the NASEM TDN intake equation [1], although it had a slightly increased mean bias, was comparable to the other equations based on the Galyean and Tedeschi [2] database. This finding no doubt reflects the fact that the database of [6] from which the NASEM [1] equation was developed included 285 of the 335 observations (i.e., 85% overlap) in the Galyean and Tedeschi [2] database.

The BR-CORTE [10] equation based on the TDN and CP intakes, which was developed from a large database of MCP measurements derived from a variety of methods, had a much greater mean bias (−93.9 g/d), indicating overprediction of MCP synthesis, and a slightly greater RMSEP than the other six equations (Table 2). Nonetheless, the r^2^ and the CCC values for the BR-CORTE [10] equation were similar to other equations, suggesting a relatively constant error of prediction over the range of the data.

The simple “ten percent” equation (Equation (3)), which was derived from the mean ratio of the MCP yield to the TDN intake in the Galyean and Tedeschi [2] database, gave results similar to the other equations derived from the same database. This finding reflects the longstanding appreciation for the importance of fermentable energy (in this case with TDN as a proxy) as a driver of the MCP yield in cattle. Moreover, the relative equivalence of Equation (3) to the other equations suggests that adjustment for different diet types (e.g., diets with low concentrations of peNDF or low-quality, high-fiber forage diets with low intakes relative to body weight and associated low passage rates) as proposed by [7] is not likely to improve accuracy and precision of MCP predictions. Nonetheless, to consider the possibility that different classes of diets might have unique characteristics that would affect the prediction of MCP synthesis, we examined how including diet classifications might affect MCP predictions. To apply this approach, we classified the data in the Galyean and Tedeschi [2] database according to diet type. Three classification groups were used to categorize the data as high-concentrate diets, medium- to high-quality forage and medium-concentrate diets, and low-quality forage diets. These groupings reflected the classification of the diets provided by the authors of the papers, along with chemical composition data provided in the original papers to evaluate components like NDF and starch. The descriptive data for the three classification groups are shown in Table 3.

Notable differences in nutrient content were evident among the classes, particularly the concentrations of TDN, digestible energy, NDF, and starch. To examine the effect of including diet class in models to predict MCP synthesis, we conducted mixed-model regression analyses with the two Galyean and Tedeschi [2] equations (the TDN intake and TDN + CP intakes) shown in Table 1, with or without the inclusion of diet classes in the model. The effect of class was not significant (*p* > 0.233) in either model nor was the interaction of class with either the TDN intake (*p* > 0.095) or intakes of TDN and CP (*p* > 0.384). Thus, despite large differences in nutrient concentrations among the three diet classes, there was no evidence that MCP predictions based on intakes for TDN and CP varied among classes. This finding presumably reflects the similarity in the ratio of MCP:TDN intake in the three diet classes (Table 3) and provides further support for the use of the simple “ten percent” calculation used in Equation (3).

Overall, the predicted MCP values from all the equations we evaluated were less precise than desired. For example, the RMSEP estimates as a percentage of the overall MCP yield mean (540.7 g/d) averaged 28.6% and 28.8% for the resampling and simulated population samples, respectively. Using a more elaborate approach than what we used for equation development, which involved predictions of ruminally degraded NDF, starch, and protein based on Michaelis–Menten enzymatic kinetics, Hanigan et al. [3] reported RMSEP values as a percentage of the mean (approximately 25%) in the same range as we observed with less complex equations. Thus, a lack of precision seems to be consistently intrinsic to MCP prediction equations.

### 3.3. Resampling vs. Sampling a Simulated MVN Population for Equation Evaluation

As noted previously, results for fit statistics and the decomposition of the MSEP were not greatly different between the resampling and simulated MVN population estimates, with very similar mean bias, RMSEP, r^2^, and CCC estimates for the two evaluation methods. Moreover, all 5% and 95% quantiles overlapped for each of the seven prediction equations, indicating no statistical differences between the two approaches. These findings suggest that sampling a simulated MVN population for equation evaluation has value as an alternative to resampling with replacement. Resampling procedures are restricted to the actual values in the sample, but the simulated MVN population approach includes values that are not in the original database. Although values from the simulated MVN population would be expected to be generally within the range of the original data, the “tails” of the population could potentially include values well outside the range of the original data. Based on the similarities of the fit statistics results for the two methods, however, it does not seem likely that using the simulated MVN population approach necessarily equates to a more robust evaluation.

## 4. How Can We Improve the Prediction of Microbial Protein Synthesis?

### 4.1. Challenges to Developing Better Equations

As we have noted, the prediction errors for currently available equations to estimate MCP synthesis in both beef and dairy cattle are high, with RMSEP values ranging from 20% to 30% of the observed mean. By comparison, equations used to predict the net energy concentrations of beef cattle diets from animal performance yield estimates that are within 8% to 10% of tabular net energy values [13]. In the context of defining the protein requirements of cattle using a metabolizable protein system, this degree of uncertainty in MCP predictions makes it challenging to estimate the dietary protein needs of cattle accurately. Given that protein is often one of the most expensive supplemental nutrients, imprecision in MCP synthesis equations has real-world consequences in terms of the cost of production and the ability to estimate animal performance accurately.

### 4.2. Inherent Variability in MCP Estimates

An underlying reason for the errors associated with MCP predictions is the inherent variability and sparsity of the data from which prediction equations are developed. As mentioned previously, MCP synthesis cannot be determined directly with practical diets, but its measurement relies on indirect approaches. The use of intestinally cannulated animals combined with markers to estimate digesta flow (e.g., at the duodenum) and other markers to estimate the contribution of MCP to the digesta is generally considered to be the optimal approach to estimate MCP synthesis. Nonetheless, the method has many challenges [14], including issues related to representative sampling and marker-related errors. The Galyean and Tedeschi [2] database of 335 observations is based on experiments with intestinally cannulated animals, with virtually all the studies using some nucleic acid (largely purine bases) analysis as a marker for microbial protein. The technique requires the collection and analysis of the ratio of the marker to crude protein in microbial cells, but many studies in the literature have assumed a value for this ratio, which likely adds to the variability of MCP estimates.

To illustrate the variability in the estimates of MCP synthesis, we drew a random sample of 20 studies from the 78 studies in the Galyean and Tedeschi [2] database. We then used the mean and standard error data provided in each paper to calculate the standard deviation and coefficient of variation (CV) for MCP synthesis. The results of this analysis are shown in Table 4.

In addition to the values for MCP synthesis and its associated standard error and CV, values for other aspects of the 20 randomly selected studies are shown to indicate the similarity of these data to the overall database. The average CV in these studies was 19.1% (median 17.3%) with a range of 6.8% to 49.4% (Table 3), indicating large variability in the estimates of MCP synthesis based on intestinal sampling. We are not aware of a comparable analysis with urinary sampling of purine derivatives to estimate MCP synthesis; however, the CV values of such estimates are likely to be as large, if not larger, mainly when spot samples of urine are used to estimate the total urine output. Given this variability, it is not surprising that MCP prediction equations are subject to large errors relative to the mean.

### 4.3. Moving Forward

So how do we move forward in developing more accurate and precise equations to predict MCP synthesis? To start, we need more estimates of MCP synthesis in cattle, but these estimates need to be obtained with consistent techniques that yield greater precision than is currently available in the literature. Although avoiding invasive surgical procedures to obtain these estimates is undoubtedly an admirable goal, the use of animals with a cannula (i.e., fistula) in the intestinal tract still seems to be the “gold standard” method. Sampling from the omasum limits surgical intervention to ruminal cannulation, and once sampling equipment and expertise are in place digesta estimates seem on par with duodenal sampling [15]. Nonetheless, differences have been noted between omasal and duodenal sampling [16]. Sampling from the reticulum offers a simpler alternative to other sites and, although limited data indicate that it shows promise compared to omasal sampling [17], much more work is needed to verify the utility of this approach. The choice of digesta flow marker(s) is another critical issue, with dual- [18] and triple- [15] marker systems generally considered to provide more representative samples of digesta than single-marker approaches [19]. Microbial markers are also a concern, with continuous ^15^N infusion likely providing more accurate estimates than those obtained with purine bases [20,21]. Obtaining representative samples of microbial matter to estimate the composition of nitrogen and microbial markers is also a critical step regardless of the microbial marker used.

Developing alternative methods to sample the digesta, estimate the digesta flow, and estimate the contribution of MCP to the digesta should be a priority moving forward, although support for the technique development is too often not a focus of funding agencies. The use of purine derivatives in urine samples deserves further attention, as does the development of accurate methods to determine the urine flow in free-ranging cattle. Lima et al. [14] suggested that omics techniques might allow us to characterize the rumen microbial population better, leading to improved approaches to estimate the contribution of MCP to both the intestinal digesta and urine samples. For example, taxonomic data combined with assembled genome data might be directly predictive of MCP synthesis using regression methods. Alternatively, taxonomic or genomic data combined with compositional analyses for various ruminal genera or the species within genera could allow for developing more accurate markers of MCP in digesta samples. Nonetheless, considerable variations in the composition of the rumen microbiome among animals fed the same diet under similar conditions [22], as well as considerable variation in the microbiome associated with various locations within the rumen [23] will no doubt complicate efforts to more accurately characterize the nature of MCP in the digesta. Ultimately, using metagenomics, metaproteomics, metataxonomics, and metatranscriptomics methodologies to study MCP synthesis as suggested by [14] will require a highly focused commitment of time and resources, with resulting data verified by existing or improved methods to estimate the digesta flow and urine volume.

Mechanistic models might provide more accurate and precise estimates of MCP synthesis than the current empirical approaches. Unfortunately, the adequacy of mechanistic models (or any other mathematical modeling approach) also depends on the reliability of the data used to develop the model coefficients and the interrelationships among variables for a wide range of dietary and animal situations. It will also depend on the availability of the variables in addition to the ones that are currently measured. For instance, the fermentable organic matter, passage rate, and degradation rate are vital variables that are believed to affect microbial growth but are not frequently measured for specific reasons. At present, the comparisons between the empirical and mechanistic models to predict MCP flows have not shown any benefits to mechanistic approaches [24], likely because mechanistic models use the same set of variables as the empirical ones. The ultimate success of modeling efforts—empirical or mechanistic, static or dynamic, deterministic or probabilistic—will require a commitment to national and international funding of a standardized approach of data collection, analysis, and interpretation among collaborators at research locations around the globe, a commitment that does not seem to attract the attention of funding agencies despite its direct impact on the environment and sustainability of beef production. Readers are referred to [25,26] for a more comprehensive discussion of the challenges and opportunities associated with mathematical modeling.

## 5. Summary and Conclusions

Despite the importance of MCP predictions in determining the protein requirements of cattle using metabolizable protein systems, the currently available MCP prediction equations have significant errors relative to the mean. Energy intake (e.g., TDN, digestible and metabolizable energy) has been consistently identified as a driver of MCP synthesis, with the supply of CP or ruminally degraded CP also being important factors. Increasing the complexity of equations by adding more independent variables, adding diet classifications, and using mechanistic vs. empirical approaches has not markedly improved the precision of predictions. This finding likely reflects the correlations among independent variables and inherently high variability in the estimates of MCP synthesis, which is measured indirectly and typically with considerable error relative to the mean. To improve MCP predictions, we will need more data for equation building, with MCP estimates that are derived from consistent and more precise methods. The development of standardized techniques to estimate the digesta flow and new methods to define the concentration of MCP in the digesta should be a focus of research if we expect to improve the accuracy and precision of MCP predictions significantly.

## Figures and Tables

**Table 1:** Extant and newly developed equations used to evaluate relationships between predicted and observed values from the Galyean and Tedeschi [2] database.

Source ^1^	Equation
*Extant*	
Galyean and Tesdeschi [2]—TDNI equation	MCP = 17.1828 + 0.09472 × TDNI
Galyean and Tedeschi [2]—TDNI and CPI equation	MCP = 37.2802 + 0.0708 × TDNI + 0.1147 × CPI
NASEM [1]—TDNI equation	MCP = 42.73 + 0.087 × TDNI
BR-CORTE [10]—TDNI and CPI equation	MCP = −53.07 + 0.3049 × CPI + 0.0908 × TDNI − 0.00000313 × TDNI ^2^
*Newly Developed*	
Equation (1) ^2^	MCP = 8.9719 + 0.07119 × OMI + 0.1050 × CPI − 0.02945 × NDFI
Equation (2) ^3^	MCP = 12.0676 + 0.06812 × OMI + 8.3864 × CP − 1.8243 × NDF
Equation (3)	MCP = 0.10 × TDNI

^1^ MCP = microbial crude protein, g/d; TDNI = total digestible nutrients intake, g/d; OMI = organic matter intake, g/d; CPI = crude protein intake, g/d, NDFI = neutral detergent fiber intake, g/d; NDF = neutral detergent fiber, % of DM; CP = crude protein, % of DM; ^2^ 95% confidence intervals for Equation (1) were: intercept (−58.2497, 76.1936; *p* = 0.791); OMI slope (0.05716, 0.08522; *p* < 0.001); CPI slope (0.05742, 0.1525; *p* < 0.001); and NDFI slope (−0.04485, −0.01405; *p* < 0.001). The root mean square error for citation-adjusted data was 49.20 g/d, with R^2^ = 0.931; ^3^ 95% confidence intervals for Equation (2) were: intercept (−83.5044, 107.64; *p* = 0.8012;); OMI slope (0.05983, 0.07641; *p* < 0.001); CP slope (4.6441, 12.1287; *p* < 0.001); and NDF slope (−3.1067, −0.5420; *p* < 0.006). The root mean square error for citation-adjusted data was 48.88 g/d, with R^2^ = 0.918.

**Table 2:** Fit statistics derived from resampling a literature database (500 resamples of 335 datapoints) or from sampling a synthetic multivariate normal population (500 samples of 335 observations per sample) derived from the Galyean and Tedeschi [2] database of cattle studies for observed vs. predicted microbial protein synthesis (g/d) using the extant equations in the literature and newly developed equations.

	Fit Statistics ^2^				MSEP, % ^3^		
Equation ^1^	Mean Bias, g/d	RMSEP	R ^2^	CCC	Mean	Slope	Random
		*Resampling Estimates*					
Galyean and Tedeschi [2]—TDNI equation							
Average	4.4	153.0	0.601	0.744	0.4	0.6	99.0
5% Quantile	−12.2	139.1	0.533	0.698	0.0	0.0	96.0
95% Quantile	20.7	167.1	0.660	0.782	1.7	2.8	100
Galyean and Tedeschi [2]—TDNI and CPI equation							
Average	4.1	147.6	0.630	0.761	0.4	0.9	98.7
5% Quantile	−11.9	134.2	0.567	0.721	0.0	0.0	95.1
95% Quantile	20.0	161.4	0.687	0.798	1.7	3.8	100
NASEM [1]—TDNI equation							
Average	21.1	155.6	0.601	0.721	2.1	2.0	95.9
5% Quantile	3.9	140.4	0.533	0.675	0.1	0.0	89.8
95% Quantile	37.5	171.1	0.660	0.761	5.2	6.0	99.5
BR-CORTE [10]—TDNI and CPI equation							
Average	−93.9	178.0	0.631	0.733	27.9	4.3	67.8
5% Quantile	−109.4	164.5	0.566	0.680	20.1	0.8	57.3
95% Quantile	−78.0	192.3	0.687	0.777	36.0	9.4	77.2
Equation (1)							
Average	3.6	144.4	0.648	0.769	0.4	1.5	98.1
5% Quantile	−11.9	130.9	0.584	0.729	0.0	0.0	93.9
95% Quantile	19.3	159.1	0.699	0.805	1.7	4.7	99.9
Equation (2)							
Average	2.8	150.2	0.630	0.733	0.3	4.3	95.4
5% Quantile	−13.3	135.9	0.569	0.692	0.0	0.7	90.0
95% Quantile	18.9	166.0	0.682	0.772	1.7	9.4	99.1
Equation (3)							
Average	−7.4	153.1	0.601	0.754	0.5	0.6	98.9
5% Quantile	−23.9	140.1	0.533	0.708	0.0	0.0	95.5
95% Quantile	9.0	165.8	0.660	0.790	2.5	2.8	100.0
		*Simulated MVN Population Estimates* ^4^					
Galyean and Tedeschi [2]—TDNI equation							
Average	3.4	153.7	0.602	0.744	0.3	0.5	99.2
5% Quantile	−11.5	142.8	0.528	0.692	0.0	0.0	97.1
95% Quantile	20.7	165.6	0.666	0.785	2.0	2.1	100
Galyean and Tedeschi [2]—TDNI and CPI equation							
Average	3.0	148.5	0.631	0.761	0.3	0.9	98.8
5% Quantile	−11.9	137.7	0.556	0.709	0.0	0.0	96.0
95% Quantile	20.6	160.0	0.692	0.799	2.0	3.2	99.9
NASEM [1]—TDNI equation							
Average	20.1	156.3	0.602	0.721	1.9	2.1	96.0
5% Quantile	4.8	145.0	0.528	0.668	0.1	0.1	91.5
95% Quantile	38.5	168.9	0.666	0.764	6.0	5.7	99.1
BR-CORTE [10]—TDNI and CPI equation							
Average	−95.5	179.5	0.636	0.737	28.4	5.0	66.6
5% Quantile	−111.5	167.0	0.565	0.682	21.0	1.7	59.0
95% Quantile	−77.9	193.7	0.696	0.784	35.8	9.2	74.2
Equation (1)							
Average	4.3	145.7	0.646	0.768	0.4	1.4	98.2
5% Quantile	−11.8	135.2	0.576	0.720	0.0	0.0	95.3
95% Quantile	20.5	156.7	0.706	0.805	2.1	4.4	99.9
Equation (2)							
Average	2.8	151.4	0.630	0.732	0.3	4.5	95.2
5% Quantile	−13.5	140.4	0.556	0.683	0.0	1.0	90.5
95% Quantile	19.8	162.4	0.689	0.771	1.8	9.3	98.9
Equation (3)							
Average	−8.4	153.8	0.602	0.754	0.6	0.4	99.0
5% Quantile	−23.4	142.1	0.528	0.700	0.0	0.0	96.7
95% Quantile	8.5	166.2	0.666	0.795	2.3	1.9	100.0

^1^ Equations evaluated are shown in Table 1; ^2^ mean bias = mean of observed values minus mean of predicted values. RMSEP = root mean square error of prediction; r^2^ = coefficient of determination; CCC = concordance correlation coefficient (the degree to which x and y pairs fall on the 45° line through the origin); ^3^ percent decomposition of the mean square error of prediction (MSEP) into mean bias, systematic (slope) bias, and random errors; and ^4^ MVN = multivariate normal.

**Table 3:** Descriptive statistics for subjective diet classifications derived from the Galyean and Tedeschi [2] database of cattle studies.

Diet Class	No.	Variable ^1^	Mean	SD ^2^	Minimum	Maximum
High-concentrate diets	148	MCP yield, g/d	577.9	241.9	218.8	1462.5
		MCP yield/TDN intake, g/g	0.10	0.02	0.05	0.16
		TDN, %	81.8	4.8	68.7	89.9
		DE, Mcal/kg	3.35	0.17	2.88	3.65
		Ether extract, %	4.4	2.0	1.4	9.6
		Crude protein, %	13.2	1.9	8.0	20.0
		Neutral detergent fiber, %	17.2	4.9	11.3	30.3
		Starch, %	53.3	7.8	30.0	71.0
		Acid detergent fiber, %	8.4	1.9	3.7	18.0
		DM intake, g/d	7259.4	2384.6	3151.0	14,500.0
		OM intake, g/d	6878.5	2229.0	2963.0	13,800.0
Low-quality forage diets	70	MCP yield, g/d	390.4	149.8	120.6	728.8
		MCP yield/TDN intake, g/g	0.11	0.04	0.05	0.19
		TDN, %	53.1	5.1	35.1	67.5
		DE, Mcal/kg	2.31	0.19	1.55	2.83
		Ether extract, %	2.4	1.5	0.9	11.2
		Crude protein, %	8.5	2.5	2.3	16.4
		Neutral detergent fiber, %	67.1	7.4	52.1	80.8
		Starch, %	1.7	3.6	0.00	13.7
		Acid detergent fiber, %	39.0	5.8	29.0	51.4
		DM intake, g/d	7277.7	2155.5	2499.5	11,389.4
		OM intake, g/d	6702.4	1963.6	2287.0	10,750.0
Medium- to high-quality forages and medium-concentrate diets	117	MCP yield, g/d	583.6	255.6	159.4	1325.0
		MCP yield/TDN intake, g/g	0.10	0.03	0.06	0.17
		TDN, %	72.5	8.1	55.8	87.9
		DE, Mcal/kg	3.02	0.29	2.40	3.57
		Ether extract, %	4.1	2.2	1.6	9.8
		Crude protein, %	14.3	2.8	6.7	22.0
		Neutral detergent fiber, %	36.7	15.3	14.7	81.8
		Starch, %	28.8	16.1	0.0	55.5
		Acid detergent fiber, %	21.1	9.1	8.8	51.0
		DM intake, g/d	8144.6	2425.9	3096.0	14,600.0
		OM intake, g/d	7583.5	2221.9	2956.7	13,687.0

^1^ MCP = microbial crude protein; TDN = total digestible nutrients; DE = digestible energy; DM = dry matter; and OM = organic matter. All dietary components are expressed on a DM basis; ^2^ SD = standard deviation.

**Table 4:** Descriptive statistics for 20 randomly selected studies (78 treatment mean observations) from the Galyean and Tedeschi [2] database used to evaluate variation associated with measurement of microbial crude protein synthesis in cattle.

Variable ^1^	Mean	SD ^2^	Minimum	Maximum	Median
*MCP measurements*					
MCP yield, g/d	579.3	274.8	158.1	1462.5	556.3
MCP yield SEM, g/d ^3^	47.9	25.9	15.6	100.0	35.0
MCP yield CV, % ^3^	19.1	10.4	6.8	49.4	17.3
*Other sample characteristics*					
MCP yield/TDN intake, g/g	0.11	0.03	0.06	0.19	0.10
TDN, %	73.3	11.8	35.1	89.3	78.2
DE, Mcal/kg	3.04	0.43	1.55	3.62	3.22
Ether extract, %	4.0	2.4	1.6	11.2	2.9
Crude protein, %	12.9	2.5	5.2	20.0	12.8
Neutral detergent fiber, %	31.1	19.2	12.2	80.8	25.3
Starch, %	37.0	19.9	0.0	68.3	41.1
Acid detergent fiber, %	17.2	12.2	6.2	51.4	11.7
DM intake, g/d	7599.4	2660.8	3130.0	13,500.0	7830.0
OM intake, g/d	7143.8	2447.7	2963.0	12,520.0	7335.0

^1^ MCP = microbial crude protein; TDN = total digestible nutrients; DE = digestible energy; DM = dry matter; and OM = organic matter. All dietary components are expressed on a DM basis; ^2^ SD = standard deviation; ^3^ SEM = standard error of the mean reported in the study; and CV = coefficient of variation (SD as a percentage of the mean) reported in the study.

## Data Availability

Data sharing is not applicable to this article.
